# Elevated Serum IgA at Onset of Type 1 Diabetes in Children

**DOI:** 10.1155/2024/7284088

**Published:** 2024-03-19

**Authors:** Amruta Thakkar, Xiaofan Huang, Johnny Wang, Kathy Hwu, Ivan K. Chinn, Charles Minard, Joud Hajjar, Maria J. Redondo

**Affiliations:** ^1^Division of Diabetes and Endocrinology, Department of Pediatrics, Texas Children's Hospital, Baylor College of Medicine, Houston, TX, USA; ^2^Baylor College of Medicine, Houston, TX, USA; ^3^Undergraduate School, Rice University, Houston, TX, USA; ^4^Division of Immunology, Allergy and Retrovirology, Department of Pediatrics, Texas Children's Hospital, Baylor College of Medicine, Houston, TX, USA; ^5^Division of Immunology, Allergy and Retrovirology, Department of Medicine, Baylor College of Medicine, Houston, TX, USA

## Abstract

**Background:**

Elevated serum IgA levels have been observed in various autoimmune conditions, including type 1 diabetes (T1D). However, whether children with T1D and elevated serum IgA have unique features has not been studied. We aimed to evaluate the prevalence and characteristics associated with elevated serum IgA at the onset of pediatric T1D.

**Materials and Methods:**

We analyzed demographic, clinical, and laboratory data retrospectively collected from 631 racially diverse children (6 months–18 years of age) with T1D who had serum IgA levels measured within 90 days of T1D diagnosis. Univariable and multivariable logistic regression models were used to identify characteristics that were significantly associated with elevated versus normal IgA.

**Results:**

Elevated serum IgA was present in 20.3% (128/631) of the children with newly diagnosed T1D. After adjusting for other variables, A1c level (*p*=0.029), positive insulin autoantibodies (IAA) (*p*=0.041), negative glutamic acid decarboxylase autoantibodies (GADA) (*p*=0.005) and Hispanic ethnicity (*p*  < 0.001) were significantly associated with elevated serum IgA. After adjustment for confounders, the odds of elevated serum IgA were significantly increased with positive IAA (OR 1.653, 95% CI 1.019–2.679), higher HbA1c (OR 1.132, 95% CI 1.014–1.268) and Hispanic ethnicity (OR 3.279, 95% CI 2.003–5.359) but decreased with GADA positivity (OR 0.474, 95% CI 0.281–0.805).

**Conclusions:**

Elevated serum IgA is present in 20.3% of the children at T1D onset and is associated with specific demographic and clinical characteristics, suggesting a unique pathogenesis in a subset of individuals. Further studies are warranted to investigate the IgA response, its role in T1D pathogenesis, and whether these associations persist over time.

## 1. Introduction

Type 1 diabetes (T1D) is one of the most common chronic diseases affecting children, with an estimated prevalence of over 2.15 per 1,000 youth less than 20 years of age in the United States [1]. Despite recent advances in care, T1D still requires lifelong management with insulin therapy, may lead to end-organ complications, and reduces life expectancy [2–5]. The increasing incidence and health burden of T1D supports the need to elucidate the mechanisms underlying its pathophysiology in order to implement preventative strategies [6]. Disease-modifying therapies that delay the onset of clinical T1D [7] or that slow down the decline in C-peptide after diagnosis are emerging [8–11]. However, the mechanism that underlie variability in response to these therapies are still poorly understood. To identify individuals who are more likely to respond and design novel interventions for those who do not, it will be important to develop biomarkers that identify the underlying pathogenic process and monitor response to treatment [12].

While selective IgA deficiency has been extensively studied [13–19], the significance of elevated serum IgA is not well known. Elevated IgA levels have been reported in various autoimmune conditions and genetic disorders of immune dysregulation [20, 21], including T1D [22–24]. In rheumatoid arthritis, elevated serum IgA was associated with more severe disease characteristics [25, 26]. In another study, diabetic complications in both type 1 and type 2 diabetes were associated with a significant increase in serum IgA concentrations [24]. However, to our knowledge, whether children with elevated IgA at the onset of T1D have differential characteristics has not been previously studied. Therefore, we aimed to compare the phenotypic characteristics of children with new onset T1D with elevated and normal serum IgA levels. Finding that elevated IgA is associated with distinct features could suggest a unique pathophysiology of their disease, which may open new opportunities for prevention and/or treatment.

## 2. Materials and Methods

This was a retrospective study of electronic medical record data of 707 children between 6 months and 18 years of age with new onset T1D diagnosed between January 2008 and February 2012 at a large, academic pediatric hospital. T1D was defined according to the ADA criteria [27]. Children without an available serum IgA measurement within 90 days since diagnosis (*n* = 76) were excluded, resulting in a sample of 631 children. The study was approved, and the requirement of informed consent was waived by the Institutional Review Board (IRB) of Baylor College of Medicine (IRB protocol H-8824).

Demographic and clinical data were collected by review of electronic medical records. Demographic information included age at diagnosis, sex, self-reported race, and ethnicity. Sex- and age-adjusted BMI percentile were based on weight and height at diagnosis. Tanner stage of pubertal development was documented by a pediatric endocrinologist. We also collected laboratory measures that are systematically obtained, per clinical protocol, from all children who present with new-onset diabetes at the time of diagnosis, which include serum glucose, hemoglobin A1c (HbA1c), pH, bicarbonate (bicarb), beta-hydroxybutyrate (BOHB), random C-peptide, islet autoantibodies (GADA, IAA, and IA-2A) and thyroid antibodies (thyroid peroxidase and thyroglobulin antibodies). In addition, this clinical protocol includes obtaining a celiac screen, which encompasses serum total IgA and tissue transglutaminase IgA (tTg IgA). Diabetic ketoacidosis (DKA) was defined by criteria per ISPAD consensus guidelines [28]. Serum IgA was measured using nephelometry as part of the celiac screen at Texas Children's Hospital Laboratory. IgA values above age-adjusted normal ranges were defined as elevated serum IgA (Table S1).

Patient characteristics were summarized by median with 25th and 75th percentiles or frequency with proportion. Demographic and clinical variables were compared between normal and high serum IgA groups using the Wilcoxon rank sum test or Pearson Chi-square test. Univariable logistic regression was used to identify baseline characteristics that were significantly associated with high IgA (vs. normal IgA). To adjust for potential confounders, multiple logistic regression models were developed which included all the significant factors from the univariable model. In the multivariable selection process, pH, BOHB, and bicarb were excluded since they were highly correlated with DKA. The stepwise selection was used to choose the best-reduced model by the Akaike information criterion. All analyses were performed using Stata 12 (StataCorp LLC, College Station, TX, USA). Statistical significance was assessed at the 0.05 level.

## 3. Results

Among the 631 children with new-onset T1D included in the analysis, the mean age at onset of T1D was 9.7 (±4.2) years, with 52% being male. The racial and ethnic distribution included 59% non-Hispanic White, 20% Hispanic, 16% non-Hispanic African American, and 5% non-Hispanic other races. Of these children, serum IgA was normal in 484 (76.7%), elevated in 128 (20.3%) and low in 19 (3.0%) of the children. Additional characteristics are described in Table 1.

**Figure 1:**
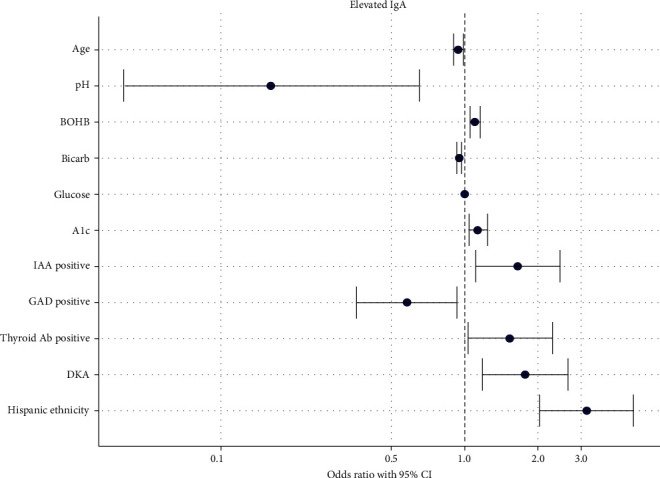
OR plot (univariable analysis): Association of significant characteristics with elevated serum IgA.

Compared to those with normal IgA (*n* = 484), children with elevated IgA were younger (median 8.9 years vs. 10.1 years, *p*=0.007), had a higher median HbA1C at diagnosis (12.4% vs. 11.8%, *p*=0.016) and were more likely to test positive for IAA (47% vs. 35%, *p*=0.013). Other comparisons of characteristics are listed in Table 2. In univariate analysis, IAA positivity and the presence of DKA at diagnosis were associated with increased odds of elevated IgA levels, with respective odds ratios of 1.650 (95% CI 1.108–2.455) and 1.769 (95% CI 1.177–2.657). Conversely, GADA positivity decreased the odds of elevated IgA levels by 42% (OR 0.578, 95% CI 0.364–0.932). Notably, Hispanic ethnicity was associated with more than a threefold increase in odds of elevated IgA (OR 3.165, 95% CI 2.026–4.927). A graphical representation of the influence of these and other variables on the odds of elevated serum IgA are shown in Figure 1 (Table S2).

**Figure 2:**
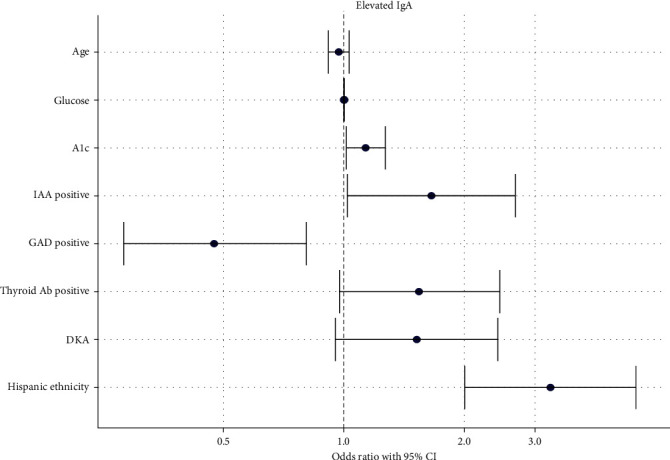
OR plot (multivariable analysis): Association of various characteristics with elevated serum IgA with adjustment for confounding variables. PH, BOHB, and bicarb were excluded from the analysis since they were highly correlated with DKA.

**Figure 3:**
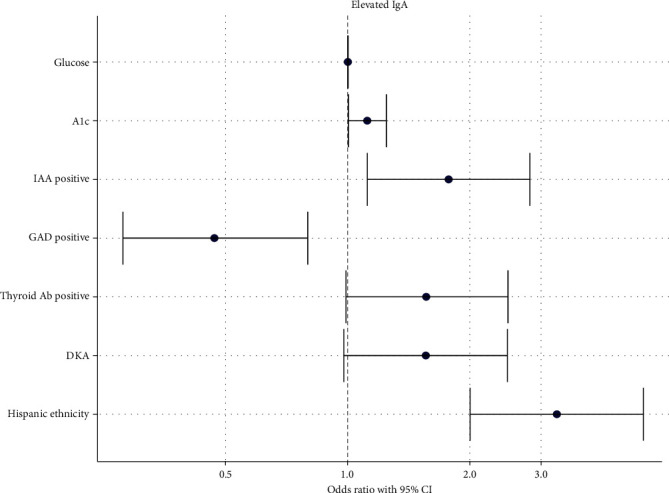
OR plot multivariable analysis (reduced model): Association of characteristics with elevated serum IgA with adjustment for confounding variables.

On multivariable logistic regression analysis, after adjusting for other variables, several factors remained associated with elevated serum IgA at diagnosis. These included A1c level (*p*=0.029), IAA positive status (*p*=0.041), GADA negative status (*p*=0.005) and Hispanic ethnicity (*p*  < 0.001). Age, glucose level at diagnosis, thyroid antibody positivity, and presence of DKA did not remain significant. Notably, the odds of having elevated serum IgA were significantly increased in children with positive IAA (OR 1.653, 95% CI 1.019–2.679), higher HbA1c (OR 1.132, 95% CI 1.014–1.268), and those of Hispanic ethnicity (OR 3.279, 95% CI 2.003–5.359). Conversely, GADA positivity was associated with a decreased odds of elevated serum IgA (OR 0.474, 95% CI 0.281–0.805) (Figure 2; Table S3). Similar results were observed in the reduced model, as depicted in Figure 3 (Table S4).

## 4. Discussion

In this study of 631 children with new onset T1D, 20.3% had elevated serum IgA levels, consistent with previous studies [24]. We found that after adjusting for confounders, elevated serum IgA was significantly associated with Hispanic ethnicity, higher A1C, higher prevalence of IAA positivity, and lower prevalence of GADA positivity.

While elevated serum IgA levels in T1D have been reported in previous publications [22–24], to our knowledge, its associations have not been previously studied. Serum IgA is produced by plasma cells in the bone marrow and marginal zone B cells [29], and is largely considered to be a neutralizing antibody due to its ability to downregulate the inflammatory response by monomeric binding to the Fc alpha receptor of immune cells [30–33]. However, studies have also found that IgA binding to pathogens can induce crosslinking of Fc alpha receptors expressed by the myeloid cells, leading to a pro-inflammatory response with phagocytosis, respiratory burst, antibody-dependent cytotoxicity, increased antigen presentation, degranulation, and cytokine release [34, 35]. Thus, inhibitory signals seem to occur in the normal physiological state when IgA titers are lower, but in pathogenic states, IgA may activate immune effector cells to carry out their functions [29, 34, 35]. Furthermore, Wilmore et al. [36] demonstrated that modulations in the microbial composition of the gut in mice resulted in heightened serum IgA concentrations and conferred IgA-mediated resistance against polymicrobial sepsis. Gut dysbiosis, characterized by alterations in the normal gut microbial composition, is increasingly being identified as a key environmental factor in the development of T1D [37–41]. Therefore, it is possible that elevated serum IgA in a subset of children with T1D may be a response to gut dysbiosis, which could be playing a role in the pathogenesis of T1D in these children. Similarly, Luopajärvi et al. [42] reported aberrant humoral immune responses, including increased serum IgA to cow's milk formula, in at-risk children who later progressed to T1D when compared with HLA-matched unaffected controls. They suggested that earlier initiation of cow's milk-based formula in infants may cause increased intestinal permeability, gut inflammation, and immune stimulation, which may be an early event in the pathogenesis of T1D.

In addition, increased serum IgA levels are known to be present in genetic disorders of immune deficiency or dysregulation. Examples include defects in *FOXP3, IKBKG, WAS, ARPC1B, GINS1, SPINK5, RASGRP1, STK4, TNFRSF6*, and *TLR8*, among others [43, 44]. Of interest, *FOXP3* encodes the nuclear forkhead box P3 transcription factor that serves as the master programmer for regulatory T cell development and function. *FOXP3*-deficient patients often develop T1D as part of their disease presentation [45, 46]. The well-known association between *IKBKG* deficiency and elevated IgA levels [47]; on the other hand, suggests that NF-*κ*B signaling also plays a crucial role in IgA production by B cells. The presence of increased IgA levels in such established immunologic disorders and recognition of elevated serum IgA levels in a significant portion of children with T1D, therefore, suggests the possibility of underlying genetic defects that impact immunity in these patients.

In our study, we observed a positive association between elevated serum IgA levels and IAA positivity versus a negative association between elevated serum IgA and GADA. In the TEDDY Study, Krischer et al. [48] observed that the appearance of IAA first tends to be associated with the HLA DR4 haplotype, while GADA, as the first autoantibody, was associated with the HLA-DR3 haplotype. The same study also showed that triggers of IAA differ from those of GADA only as a first-appearing autoantibody [49]. In previous studies, children at risk for T1D who developed IAA as their first positive autoantibody were younger and progressed faster to clinical T1D than children whose first autoantibody was GADA [50–52]. Our observation that, at the onset of pediatric T1D, elevated serum IgA is associated with IAA positivity and GADA negativity supports the hypothesis that these two islet autoantibodies mark differential pathological mechanisms in the development of T1D [48–50, 52]. Independently from potential confounders, we observed that children with elevated IgA also had higher A1c, which could reflect higher glucose levels as a result of lower residual insulin secretion and/or longer duration of hyperglycemia before diagnosis, particularly because the glucose was not different between the two groups,

Finally, we found that Hispanic ethnicity was significantly associated with elevated IgA. As earlier studies have reported a lower risk of progression to T1D, particularly in the earlier stages of islet autoimmunity in Hispanic children [6], and Hispanic children had a higher prevalence of elevated IgA in the current study, and this may signify that these elevated levels may be involved in the development of T1D in those Hispanic children who developed the disease.

Our study had several limitations. Inherent to a retrospective observational review, this study is capable of identifying associations but cannot confirm causality. Additionally, cross sectional rather than longitudinal data was obtained, which limited our ability to observe IgA changes over time and its potential association with T1D course and severity. Although IgA levels at diagnosis were accounted for, the duration of positivity prior to diagnosis was not available in this study, which could have supported our hypothesis. The clonality of IgA and serum levels of other antibodies were also not available in our study. Despite these limitations, our study did detect a clear association between elevated serum IgA and several characteristics that deserve to be further evaluated. The strengths of our study included a large, racially and ethnically diverse, well-characterized pediatric cohort with real-world data reflective of the general population.

In conclusion, we identified elevated serum IgA levels in 20% of children at T1D diagnosis, finding significant association with Hispanic ethnicity, positivity for IAA, higher HbA1c, and Hispanic ethnicity. These findings may suggest a unique pathogenic mechanism leading to the development of T1D. Further studies aimed at investigating systemic IgA responses before and after the clinical diagnosis of T1D may advance our understanding of the natural history of T1D with the ultimate goal to develop strategies to prevent T1D and its progression.

## Figures and Tables

**Table 1:** Characteristics of children with new onset T1D included in the analysis (*n* = 631).

Characteristics at diagnosis	
Serum IgA levels % (*N*)	
Normal	76.7% (484)
High	20.3% (128)
Low	3.0% (19)
Male: % (*N*)	52% (330)
Age (years)	9.9 (6.6–12.8)
IgA at diagnosis (mg/dL)	159 (108–218)
Pubertal development stage: % (*N*)	
Prepubertal	59% (343)
Race/ethnicity: % (*N*)	
Non-Hispanic White race	59% (362)
Hispanic ethnicity (any race)	20% (124)
African American race	16% (96)
Asian race	4.0% (25)
Mixed race	1.0% (7)
Glucose (mg/dL)	372 (270–508)
HbA1c at diagnosis (%)	11.8 (10.4–13.5)
C-peptide, random (ng/mL)	0.4 (0.28–0.71)
pH	7.33 (7.21–7.38)
BOHB (mmol/L)	4.1 (1.4–7.8)
Bicarbonate (mmol/L)	19.0 (10.2–24.0)
DKA: % (*N*)	36% (214)
IA-2A (U/mL)	8.4 (1.5–23.0)
IA-2A positivity: % (*N*)	79% (494)
GADA (IU/mL)	5.2 (0.93–29.5)
GADA positivity: % (*N*)	82% (511)
Number of positive islet autoantibodies: % (*N*)	
0	4% (22)
1	21% (133)
2	49% (307)
3	26% (159)
IAA positivity: % (*N*)	36% (228)
IAA at diagnosis (U/mL)	0.3 (0.3–1.1)
tTG IgA positivity: % (*N*)	11% (69)
tTG IgA (U/mL)	4 (2–9)
Thyroid antibody positivity: % (*N*)	51% (316)

*a* (b − c) represents the median *a*, the lower quartile *b*, and the upper quartile *c* for continuous variables.

**Table 2:** Comparison of clinical and laboratory characteristics in children with new onset T1D with normal vs. elevated serum IgA.

Characteristics at diagnosis of T1D	Normal IgA (*N* = 484)	High IgA (*N* = 128)	*p*-Value
Male: % (*N*)	51% (245)	58% (74)	0.15
Median age (years)	10.1 (6.9–13.0)	8.9 (5.0–11.8)	**0.007**
IgA at diagnosis (mg/dL)	143 (101–186)	300 (226–358)	**<0.001**
Pubertal development stage: % (*N*)	—	—	0.64
Prepubertal	58% (261)	65% (75)	—
Race/ethnicity: % (*N*)	—	—	**<0.001**
Non-Hispanic White	63% (302)	40% (48)	—
Hispanic	16% (76)	38% (45)	—
African American	16% (74)	17% (21)	—
Asian	4% (18)	5% (6)	—
Mixed	1% (7)	0% (0.00)	—
Glucose (mg/dL)	356 (256–495)	416 (341–548)	**<0.001**
HbA1c (%)	11.8 (10.2–13.4)	12.4 (10.8–13.9)	**0.016**
C-peptide, random (ng/mL)	0.42 (0.26–0.71)	0.4 (0.30–0.68)	0.27
pH	7.34 (7.22–7.38)	7.29 (7.16–7.36)	**<0.001**
BOHB (mmol/L)	3.8 (1.2–7.5)	6.4 (3.0–8.6)	**<0.001**
Bicarbonate (mmol/L)	20 (11.2–25)	15 (8.5–21.5)	**<0.001**
DKA: % (*N*)	33% (153)	47% (57)	**0.006**
IA-2A levels (U/mL)	8.6 (1.4–24.4)	8.6 (2.1–20.8)	0.93
IA-2A positivity: % (*N*)	79% (377)	83% (105)	0.23
GADA levels (IU/mL)	5.20	4.80	0.36
GADA positivity: % (*N*)	83% (402)	74% (93)	**0.021**
IAA (U/mL)	0.3 (0.3–0.9)	0.4 (0.3–2.4)	**0.038**
IAA positivity: % (*N*)	35% (167)	47% (59)	**0.013**
Number of positive islet autoantibodies: % (*N*)	—	—	0.82
0	4% (17)	2% (3)	—
1	22% (103)	19% (24)	—
2	49% (235)	50% (63)	—
3	26% (122)	28% (35)	—
tTG IgA positivity: % (*N*)	10% (50)	15% (19)	0.14
tTG IgA levels (U/mL)	4 (2–8)	7 (4–12)	**<0.001**
Thyroid antibody positivity: % (*N*)	49% (229)	59% (74)	**0.037**

*a* (*b* − c) represents the median *a*, the lower quartile *b*, and the upper quartile *c* for continuous variables. Statistically significant *p*-values in bold (*p*  < 0.05).

## Data Availability

Data supporting the results of this study are available on request. Please contact Maria Redondo: redondo@bcm.edu for data requests.

## Supplementary Materials

Supplementary Materials Table S1: Texas Children's Laboratory IgA reference range. Table S2: univariable analysis. Table S3: multivariable analysis (full model). Table S4: multivariable analysis (reduced model).
