# Supplementary material for: Elevated Serum IgA at Onset of Type 1 Diabetes in Children
Source: Pediatr Diabetes. 2024 Mar 19;2024:7284088. doi: 10.1155/2024/7284088 (PMC12016712; doi:10.1155/2024/7284088)
Supplement: Supplementary Materials — Table S1: Texas Children's Laboratory IgA reference range. Table S2: univariable analysis. Table S3: multivariable analysis (full model). Table S4: multivariable analysis (reduced model). [file 7284088.f1.docx]

**Supplementary tables:**

**Supplementary Table S1: Texas Children’s Laboratory IgA Reference Range:**

| **Age Group** | **Normal Range** |
| --- | --- |
| >= 6Months and < 9Months | >= 10.0 and < 85.0MG/DL |
| >= 9Months and < 1Years | >= 13.0 and < 100.0MG/DL |
| >= 1Years and < 2Years | >= 13.0 and < 116.0MG/DL |
| >= 2Years and < 3Years | >= 21.0 and < 150.0MG/DL |
| >= 3Years and < 5Years | >= 22.0 and < 146.0MG/DL |
| >= 5Years and < 8Years | >= 32.0 and < 191.0MG/DL |
| >= 8Years and < 10Years | >= 42.0 and < 223.0MG/D |
| >= 10Years and < 999Years | >= 66.0 and < 295.0MG/DL |

**Supplementary Table S2: Univariable analysis:**

| **Characteristic** | **Odds Ratio** | **p-value** |
| --- | --- | --- |
| Age (years) | 0.941 [0.898-0.986] | **0.012** |
| pH | 0.161 [0.041-0.647] | **0.009** |
| BOHB (mmol/L) | 1.099 [1.047-1.155] | **<0.001** |
| Bicarbonate (mmol/L) | 0.950 [0.925-0.975] | **<0.001** |
| Glucose (mg/dL) | 1.001 [1.001-1.002] | **<0.001** |
| A1c at diagnosis | 1.130 [1.035-1.237] | **0.006** |
| IAA positivity at diagnosis % (N) | 1.650 [1.108-2.455] | **0.013** |
| GAD positivity at diagnosis % (N) | 0.578 [0.364-0.932] | **0.021** |
| Thyroid antibody positivity: % (N) | 1.527 [1.026-2.286] | **0.038** |
| DKA: % (N) | 1.769 [1.177-2.657] | **0.005** |
| Hispanic % (N) | 3.165 [2.026-4.927] | **<0.001** |

**Supplementary Table S3: Multivariable analysis (full model):**

| **Characteristic** | **Odds Ratio** | **p-value** |
| --- | --- | --- |
| Age | 0.970 [0.914-1.030] | 0.329 |
| Glucose | 1.001 [0.999-1.002] | 0.105 |
| HbA1c | 1.132 [1.014-1.268] | **0.028** |
| IAA positivity % (N) | 1.653 [1.019-2.679] | **0.04** |
| GAD positivity % (N) | 0.474 [0.281-0.805] | **0.005** |
| Thyroid antibody positivity % (N) | 1.539 [0.975-2.451] | 0.066 |
| DKA % (N) | 1.520 [0.952-2.423] | 0.078 |
| Hispanic % (N) | 3.279 [2.003-5.359] | **<0.001** |

** In the multiple model, pH, BOHB, and bicarb were excluded since they were highly correlated with DKA.*

**Supplementary Table S4: Multivariable analysis (Reduced model):**

| **Characteristic** | **Odds Ratio** | **p-value** |
| --- | --- | --- |
| Glucose | 1.001 [0.999-1.002] | **0.075** |
| HbA1c | 1.117 [1.004-1.245] | **0.042** |
| IAA positivity | 1.774 [1.118-2.814] | **0.014** |
| GAD positivity | 0.469 [0.279-0.797] | **0.004** |
| Thyroid antibody positivity | 1.562 [0.990-2.484] | **0.056** |
| DKA | 1.558 [0.978-2.477] | 0.06 |
| Hispanic | 3.279 [2.004-5.455] | **<0.001** |

**A reduced model with glucose, HBA1c positivity, IAA positivity, GADA positivity, Thyroid antibodies, presence of DKA, and Hispanic ethnicity were selected by stepwise selection by AIC.*
